# Comparing the adverse clinical outcomes in patients with non-insulin treated type 2 diabetes mellitus and patients without type 2 diabetes mellitus following percutaneous coronary intervention: a systematic review and meta-analysis

**DOI:** 10.1186/s12872-016-0422-0

**Published:** 2016-11-25

**Authors:** Nuo Li, Ye-Gui Yang, Meng-Hua Chen

**Affiliations:** Institute of Cardiovascular Diseases, the First Affiliated Hospital of Guangxi Medical University, Nanning, Guangxi 530027 People’s Republic of China

**Keywords:** Non-insulin treated diabetes mellitus, Percutaneous coronary intervention, Major adverse cardiac events, Stent thrombosis, Clinical outcomes

## Abstract

**Background:**

Several studies showed Type 2 Diabetes Mellitus (T2DM) to be associated with worse adverse clinical outcomes compared to non-T2DM (NDM) following Percutaneous Coronary Intervention (PCI). In addition, patients with insulin-treated T2DM (ITDM) showed worse clinical outcomes compared to patients with non-insulin treated T2DM (NITDM). Since NITDM and NDM have seldom been systematically analyzed, this study aimed to compare the short and long term adverse clinical outcomes observed in patients with NITDM and patients without T2DM following PCI.

**Methods:**

Medline/PubMed, EMBASE and the Cochrane library were searched for Randomized Controlled Trials (RCTs) and observational studies comparing patients with (including ITDM and NITDM) and without T2DM following PCI. Endpoints included adverse clinical outcomes reported during a short and a long term follow up period. Odd Ratios (OR) and 95% Confidence Intervals (CI) in accordance with either a fixed or a random effects model appropriately, were calculated and the pooled analyses were performed with RevMan 5.3.

**Results:**

Twelve studies consisting of a total number of 52,451 patients (14,863 NITDM and 37,588 NDM) were included. Patients with NITDM were found to have significantly higher short-term Major Adverse Cardiac Events (MACEs) and mortality with OR: 1.63, 95% CI (1.17, 2.27); *P* = 0.004 and OR: 1.71, 95% CI (1.40, 2.10), *P* < 0.00001 respectively and higher long-term MACEs and mortality with OR: 1.25, 95% CI (1.12, 1.40), *P* = 0.0001 and OR: 1.32, 95% CI (1.19, 1.47), *P* < 0.00001 respectively compared to NDM following PCI. In addition, compared to NDM, long-term Target Vessel Revascularization (TVR) and Target Lesion Revascularization (TLR) were significantly higher in the NITDM group with OR: 1.36, 95% CI (1.18, 1.56), *P* < 0.0001 and OR: 1.32, 95% CI (1.10, 1.59), *P* = 0.003 respectively. However, even if an increased long-term stent thrombosis was observed in the NITDM group with OR: 1.13; 95% CI (0.91, 1.40), *P* = 0.28, the result was insignificant.

**Conclusion:**

Short and long term MACEs and mortality were significantly higher in patients with NITDM compared to patients without diabetes following PCI. Revascularization also significantly favored patients without T2DM. However, stent thrombosis was not significantly different.

## Background

Patients with Type 2 Diabetes Mellitus (T2DM) have worse clinical outcomes compared to patients without T2DM (NDM) following Percutaneous Coronary Intervention (PCI). For example, the PRESTO trial showed that despite advances in interventional techniques, diabetes mellitus remained a significant predictor of adverse clinical events after PCI [1, 2]. Later on, it was shown that patients with insulin-treated T2DM (ITDM) had even worse adverse outcomes compared to patients with non-insulin treated T2DM (NITDM). To further illustrate this point, the FREEDOM trial showed a significantly higher rate of Major Adverse Cardiac Effects (MACEs) in those diabetic patients who were on insulin therapy following PCI compared to those patients without insulin therapy [2]. It is clear that patients without T2DM when compared to patients with T2DM and patients with NITDM compared to ITDM have lower adverse clinical events following PCI. Since NITDM and NDM have seldom been systematically analyzed, this study aimed to compare the short and long term adverse clinical outcomes observed in patients with NITDM and patients without T2DM following PCI.

## Methods

### Data sources and search strategy

Studies including Randomized Controlled Trials (RCTs) and observational studies were searched from Medline/PubMed, EMBASE and the Cochrane databases using the words ‘diabetes mellitus and percutaneous coronary intervention/PCI’ or ‘insulin-treated and non-insulin treated diabetes mellitus and PCI’. The term ‘angioplasty’ and the abbreviations ‘T2DM and DM’ were also used. Reference lists of relevant publications obtained were also checked for suitable articles. Our search began on 2nd May 2015 and ended on 30th September 2015. This search was restricted only to articles which were published in English language.

### Inclusion and exclusion criteria

Studies were included if:

- They were randomized trials and observational studies comparing the adverse outcomes between T2DM (including ITDM and NITDM) and NDM following PCI.
- They were published during or after the year 2006 (from the year 2006 to 2015).

Studies were excluded if:

- Data concerning patients with NITDM could not be retrieved from these diabetic patients.
- Patients without T2DM were not included as the control.
- Adverse clinical outcomes were not reported among their endpoints.
- They were meta-analyses, case studies or letter to editors.
- They were published before the year 2006.
- They were duplicates.

### Definitions, outcomes and follow up periods

NITDM were defined as patients who were at an earlier stage of T2DM, or did not have any diabetic complications or patients with a good control of their blood glucose levels, therefore not requiring insulin therapy as treatment. These patients were either on a diet control or on oral hypoglycemic agents.

The adverse clinical outcomes were:

- **Mortality**: defined as all-cause mortality including cardiac and non-cardiac deaths.
- **MACEs**: were defined as death of cardiac or procedure-related origin, myocardial infarction, and/or, revascularization following coronary stents implantation. Due to limited outcomes reported, major adverse cardiac and cerebrovascular events (MACCEs) were considered in the same category as MACEs and analyzed in this study.
- Target lesion revascularization (TLR) and Target vessel revascularization (TVR)
- **Myocardial infarction** (**MI**): was defined as re-infarction which occurred in these patients after PCI. Any type of MI was relevant.
- **Stent thrombosis** (**ST**): Any type of ST was considered acceptable during this analysis.

**Short term follow**-**up period**: was defined as a follow-up period of less than 1 year.

**Long term follow**-**up period**: was defined as a follow up at or during one or more years (≥ one year).

### Data extraction and quality assessment

Eligible studies were independently assessed by NL, YJY and MHC. The types of study and patients’ baseline characteristics, the total number of patients with NITDM and without T2DM respectively, the year of publication, the clinical outcomes (MACEs, death, ST, TVR, TLR, MI) and the follow up duration were systematically extracted. Any disagreement which followed was resolved by consensus. Cochrane Collaboration was considered during bias risk assessment [3].

### Methodological quality and statistical analysis

Preferred Reporting Items for Systematic Reviews and Meta-Analyses statement was followed in this type of research article [4]. The Cochrane Q-statistic test (*p* value ≤ 0 · 05 was considered statistically significant) and the *I* ^2^-statistic test (*I* ^2^ value of 0% indicated a very low heterogeneity) were used to assess heterogeneity. An *I* ^2^ value less than 50% indicated the use of a fixed effects model, whereas a random effects model was used if *I* ^2^ was greater than 50%. Funnel plots were used to assess publication bias.

We calculated Odd Ratios (OR) with 95% Confidence Intervals (CIs). The pooled analyses were performed with RevMan 5.3 software. All the authors had access to the data which were used in this study. Ethical approval was not necessary for this meta-analysis.

## Results

### Study selection

**Fig. 1:**
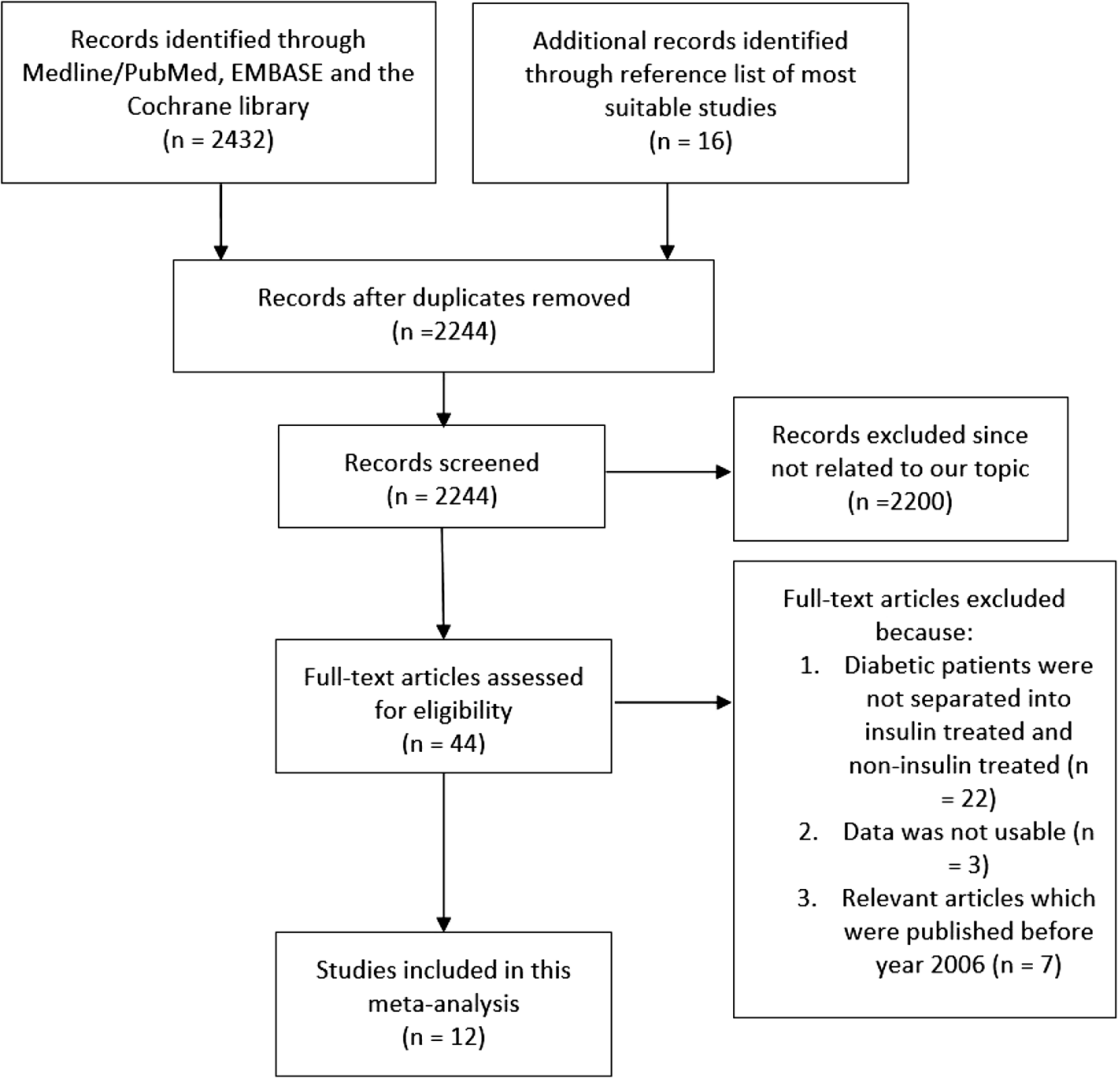
Flow diagram representing the study selection

Two thousand four hundred and thirty-two articles were identified by titles and abstracts obtained from Medline/PubMed, EMBASE and the Cochrane library. An additional 16 articles were identified from the reference lists of suitable articles. After eliminating the duplicate studies, further articles were excluded since they were not relevant to the topic of this research. Forty-four full-text articles were assessed for eligibility. Another 32 articles were excluded for the following reasons: data for patients with NITDM could not be retrieved, data were not usable or the studies were published before the year 2006. Finally, 12 articles [5–16] were selected for this meta-analysis. The flow chart showing study selection has been illustrated in Fig. 1.

Table 1 shows the clinical outcomes reported in each of the 12 studies included.

**Table 1:** Outcomes reported in each study

Study	Reported Outcomes
Kappetein 2013 [5]	MACCE, Death, MI, Revascularization, Stent thrombosis
Kereiakes 2010 [6]	MACEs, MI, Stent thrombosis
Kirtane 2008 [7]	Death, MI, TLR, TVR, Stent thrombosis
Kirtane 2009 [8]	MACEs, Death, MI, TLR, TVR, Stent thrombosis
Kumar 2007 [9]	MACEs, Death, MI, TLR, Stent thrombosis
Stone 2011 [10]	MACEs, Death, MI, TLR, Stent thrombosis
Tada 2011 [11]	Death, MI, TLR, Stent thrombosis
Thukkani 2015 [12]	Death
Witzenbichler 2011 [13]	MACEs, MI, Death, TVR, Stent thrombosis
Massalha 2015 [14]	Death
Jain 2010 [15]	MACEs, MI, TLR, TVR, Stent thrombosis
Silber 2013 [16]	Death, MACEs, TLR, TVR, Stent thrombosis

Abbreviations: *MACEs* major adverse cardiac events, *MI* myocardial infarction, *TVR* target vessel revascularization, *TLR* target lesion revascularization

### General features of the studies included in this analysis

Table 2 represents the general features including the total number of patients in the NITDM and the NDM groups respectively, the type of study, and the follow-up periods of each of the studies included in this analysis.

**Table 2:** General features of the included studies

Study	No of NITDM	No of NDM	Type of study	Follow up
Kappetein 2013 [5]	142	672	RCT	5 years
Kereiakes 2010 [6]	826	2467	RCT	1 year
Kirtane 2008 [7]	562	2686	RCT	4 years
Kirtane 2009 [8]	333	1071	RCT	1 year
Kumar 2007 [9]	182	541	OS	9 months
Stone 2011 [10]	1375	4911	RCT	2 years
Tada 2011 [11]	3404	6378	OS	3 years
Thukkani 2015 [12]	4862	7990	OS	≤12 months > 12
Witzenbichler 2011 [13]	434	3006	RCT	30 days, 1 year
Massalha 2015 [14]	196	694	OS	5 years
Jain 2010 [15]	1919	5269	OS	1 year
Silber 2013 [16]	628	1903	RCT	2 years

Abbreviations: *NITDM* non-insulin treated diabetes mellitus, *NDM* non-diabetes mellitus, *RCT* randomized controlled trials, *OS* observational studies

As shown in Table 2, this meta-analysis included a total number of 52,451 patients among which, 14,863 were NITDM patients, whereas 37,588 patients were NDM.

### Baseline characteristics

Data reporting the mean age of the patients in years, the percentage of patients with male gender, with co-morbidities such as hypertension, dyslipidemia and the percentage of patients who were current smokers were listed in Table 3. According to Table 3, no significant differences were observed in the baseline characteristics between these two groups of patients (NITDM and NDM).

**Table 3:** Baseline features of the included studies

Study	Age (years)	Males (%)	HT (%)	Ds (%)	Cs (%)
	NI/NDM	NI/NDM	NI/NDM	NI/NDM	NI/NDM
Kappetein 2013 [5]	65.4/65.0	71.0/79.9	70.0/65.0	82.0/77.0	16.0/22.0
Kereiakes 2010 [6]	63.3/63.4	63.3/70.0	87.0/71.9	82.5/72.6	18.3/24.0
Kirtane 2008 [7]	63.0/62.1	64.7/75.0	82.1/64.5	74.0/69.6	18.4/24.9
Kirtane 2009 [8]	64.0/63.3	60.4/71.0	90.6/76.7	87.1/81.4	54.1/64.8
Kumar 2007 [9]	67.0/66.0	67.0/73.0	93.0/77.0	92.0/80.0	8.0/14.0
Stone 2011 [10]	63.8/63.0	63.2/71.3	83.1/62.5	79.4/64.0	19.6/27.1
Tada 2011 [11]	67.9/68.8	76.0/76.0	78.0/73.0	−	21.0/20.0
Thukkani 2015 [12]	64.3/64.2	98.5/98.3	96.4/88.5	82.9/80.0	36.7/48.7
Witzenbichler 2011 [13]	64.5/59.6	73.4/77.2	72.3/49.8	60.3/39.7	56.8/64.9
Massalha 2015 [14]	63.0/58.0	71.0/84.0	65.0/41.0	−	22.0/23.0
Jain 2010 [15]	64.9/62.3	71.8/80.2	77.5/63.7	−	18.0/25.4
Silber 2013 [16]	65.5/63.5	70.4/74.4	86.0/73.1	86.0/76.0	18.6/22.1

Abbreviations: *HT* hypertension, *Ds* dyslipidemia, *Cs* current smoker, *NI*: non-insulin treated diabetics, *NDM* non-diabetics

### Results of this meta-analysis

A total number of 4163 patients were analyzed for MACEs and MI respectively and 17,015 patients were analyzed for mortality during the short-term follow-up period.

**Fig. 2:**
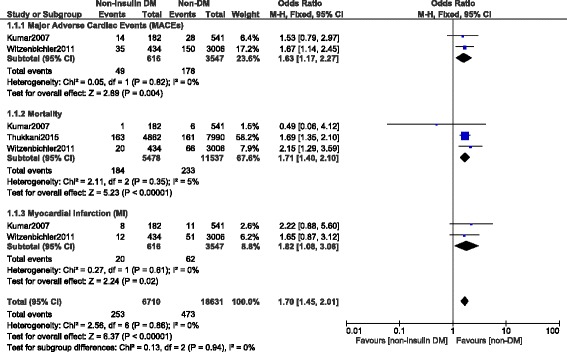
Short term adverse clinical outcomes observed between non-insulin treated T2DM and non-T2DM

This analysis showed a significantly higher rate of MACEs and MI in the NITDM group with OR: 1.63, 95% CI (1.17, 2.27); *P* = 0.04 and OR: 1.82, 95% CI (1.08, 3.06); *P* = 0.02 when compared to NDM respectively during the short term follow up period following PCI. Compared to NDM, the mortality rate was also significantly higher in the NITDM group with OR: 1.71, 95% CI (1.40, 2.10), *P* < 0.00001. Results representing the short-term outcomes were illustrated in Fig. 2. Table 4 summarized the result for the short-term follow up period.

**Table 4:** Results for the short-term analysis

Analyzed Outcomes	No of studies analyzed	Total no o of patients (*n*)	OR (Odd ratio with 95% CI)	*P* value	*I* ^2^ (%)
MACEs	2	4163	1.63 (1.17, 2.27)	0.004	0
Death	3	17,015	1.17 (1.40, 2.10)	<0.00001	5
MI	2	4163	1.82 (1.08, 3.06)	0.02	0

Abbreviations: *MACEs* major adverse cardiac effects, *MI* myocardial infarction

For the long-term follow up period, a total number of 21,465 patients were analyzed for MACEs, 37,756 patients were analyzed for mortality, 31,964 patients were analyzed for MI, 30,388 patients were analyzed for TLR, 14,320 patients were analyzed for TVR and 36,900 patients were analyzed for ST.

**Fig. 3:**
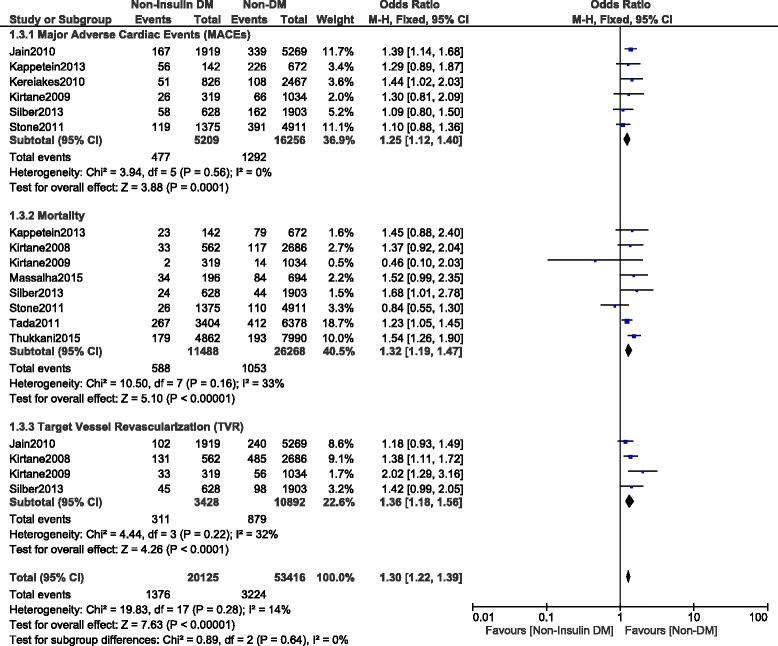
Long term adverse clinical outcomes observed between non-insulin treated T2DM and non-T2DM (part 1)

**Fig. 4:**
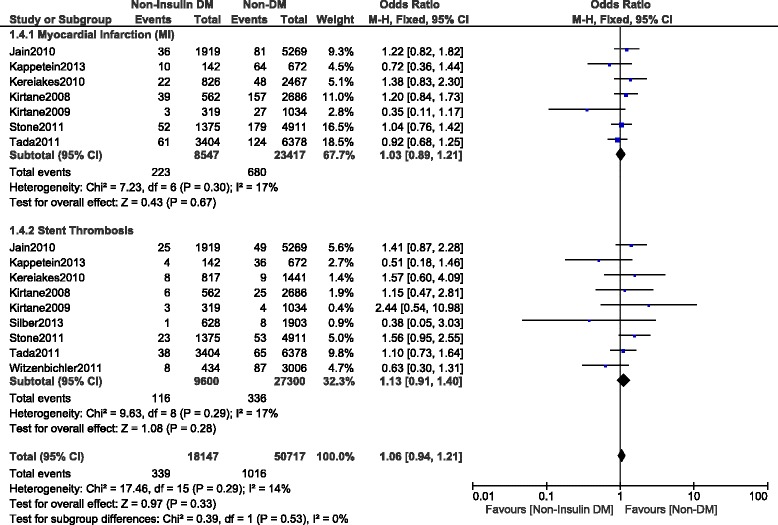
Long term adverse clinical outcomes observed between non-insulin treated T2DM and non-T2DM (part 2)

**Fig. 5:**
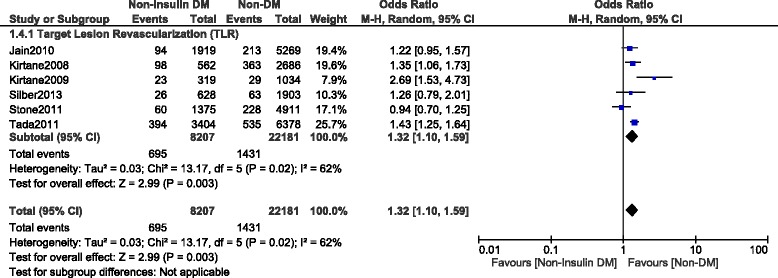
Long term Target Vessel Revascularization observed between non-insulin treated T2DM and non-T2DM

This current long-term analysis showed a significantly higher MACEs and mortality in the NITDM group with OR: 1.25, 95% CI (1.12, 1.40), *P* = 0.0001 and OR: 1.32, 95% CI (1.19, 1.47), *P* < 0.00001 respectively, and a significantly higher rate of TLR and TVR with OR: 1.32, 95% CI (1.10, 1.59), *P* = 0.003 and OR: 1.36, 95% CI (1.18, 1.56), *P* < 0.0001 respectively when compared to NDM. MI was similarly manifested with OR: 1.03, 95% CI (0.89, 1.21); *P* = 0.67. However, compared to NDM, even if the long-term ST was higher in the NITDM group, the result was not statistically significant with OR: 1.13, 95% CI (0.91, 1.40), *P* = 0.28. Results for the long-term follow up period were illustrated in Figs. 3, 4 and 5. Table 5 summarized the results for this long-term follow up period.

**Table 5:** Results for the long-term analysis

Analyzed Outcomes	No of studies analyzed	Total no of patients (n)	OR (Odd ratio with 95% CI)	*P* value	*I* ^2^ (%)
MACEs	6	21,465	1.25 (1.12, 1.40)	0.0001	0
Death	8	37,756	1.32 (1.19, 1.47)	<0.00001	33
MI	7	31,964	1.03 (0.89, 1.21)	0.67	17
TLR	6	30,388	1.32 (1.10, 1.59)	0.003	62
TVR	4	14,320	1.36 (1.18, 1.56)	<0.0001	32
ST	9	36,900	1.13 (0.91, 1.40)	0.28	17

Abbreviations: *MACEs* major adverse cardiac effects, *MI* myocardial infarction, *TLR* target lesion revascularization, *TVR* target vessel revascularization, *ST* stent thrombosis

**Fig. 6:**
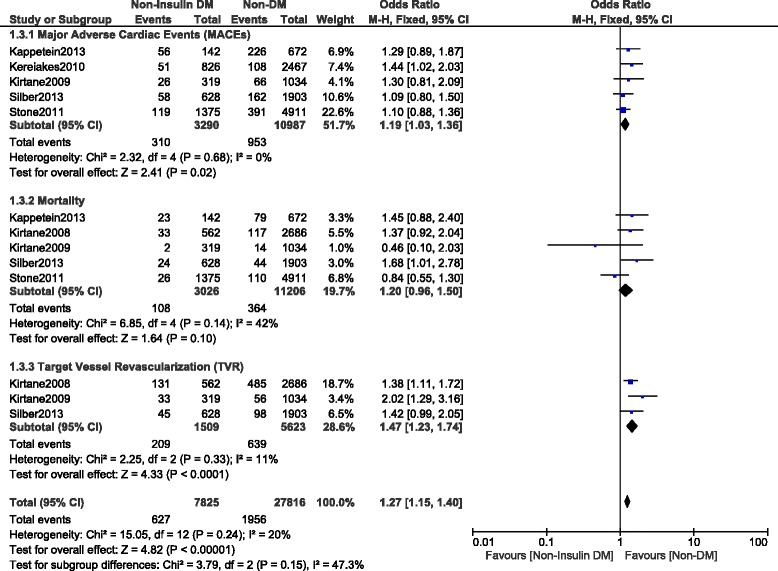
Long term adverse clinical outcomes observed between non-insulin treated T2DM and non-T2DM using only randomized patients (part 1)

**Fig. 7:**
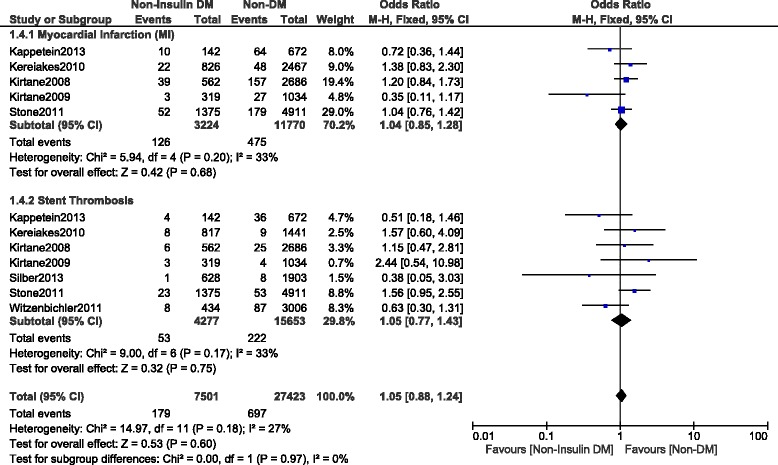
Long term adverse clinical outcomes observed between non-insulin treated T2DM and non-T2DM using only randomized patients (part 2)

A separate analysis was conducted (excluding observational studies) involving only randomized trials. The results showed long term MACEs and TVR to be significantly higher in the NITDM group with OR: 1.19, 95% CI (1.03, 1.36); *P* = 0.02 and OR: 1.47, 95% CI (1.23, 1.74); *P* < 0.0001 respectively when compared to NDM. However, even if mortality was higher in the NITDM group with OR: 1.20, 95% CI (0.96–1.50); *P* = 0.10, the result was not statistically significant. These results have been shown in Fig. 6. In addition, long term MI and ST were also not significantly different between these 2 groups with OR: 1.04, 95% CI (0.85, 1.28); *P* = 0.68 and OR: 1.05, 95% CI (0.77, 1.43); *P* = 0.75 respectively. These results have been shown in Fig. 7.

**Fig. 8:**
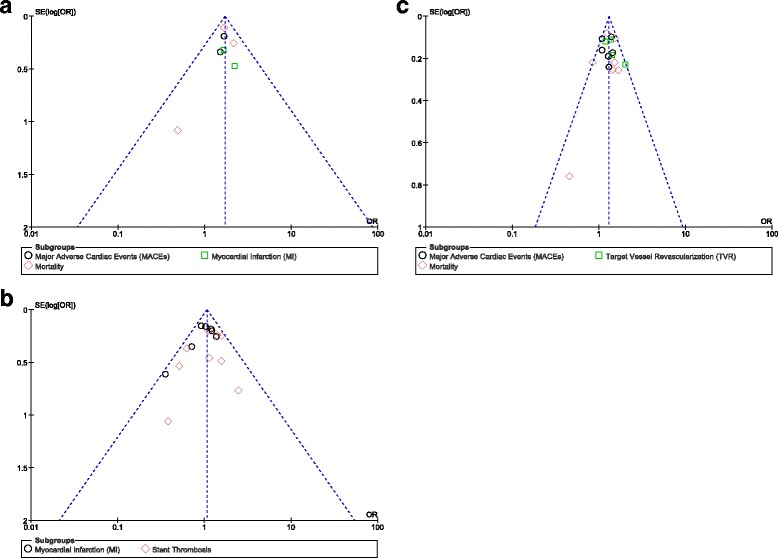
**a**–**c**: Funnel plots showing publication bias

For the included studies, publication bias was visual estimated through funnel plots. Little evidence of publication bias was observed for the included studies that assessed all clinical endpoints (Fig. 8a–c).

## Discussion

Many studies have shown T2DM to be independently associated with increased adverse clinical outcomes following PCI compared to patients without T2DM. Other studies have shown the adverse complications to be significantly higher in patients with ITDM compared to patients with NITDM. We aimed to compare the clinical outcomes between NITDM and NDM in order to know whether they have similar outcomes or not following PCI.

Results of this analysis showed significantly higher long and short term mortality and MACEs in the NITDM group compared to the NDM group. MI was similarly manifested whereas revascularization was significantly higher in patients with T2DM. Moreover, even if ST was higher in patients with NITDM compared to patients without T2DM, the result was not statistically significant in this current study.

Similar to this current analysis, the study published by Kappetein et al. also showed significantly higher MACEs and repeated revascularization (TLR and TVR) in patients with NITDM compared to those without T2DM following PCI [5]. Moreover, the PRESTO Trial which included 75% of patients with T2DM not on insulin therapy, also showed T2DM to be a significant predictor of adverse outcomes after PCI compared to NDM. Death in these patients with T2DM was reported as 2.1% compared to those patients without T2DM with only 0.9%. TVR was also higher in the T2DM group compared to the NDM group (17.9% versus 12.8%). This PRESTO Trial showed that compared to NDM, patients with T2DM had an advanced age, were mostly female patients and the majority had a history of heart failure and lower ejection fraction. These patients with T2DM were mainly overweight and obese, and had a high rate of co-morbidities [1].

The SORT OUT IV Trial also showed T2DM to be associated with a significantly higher rate of MACEs following PCI (13% in DM versus 6.4% in NDM). However, this result included patients with both ITDM and NITDM [17]. This current analysis showed long term ST to favor the NDM group without any statistical significance. However, the study published by Jensen et al. in 2010 showed T2DM to be associated with an increased risk of definite ST compared to NDM after PCI. But their result included patients with both ITDM and NITDM [18] whereas this current analysis only involved diabetic patients without insulin therapy.

This current study only analyzed patients with NITDM. However, several other studies compared patients with ITDM and NITDM have shown the former to be associated with even worse adverse outcomes after PCI. For example, results from the FREEDOM Trial showed that in patients with diabetes and multi-vessel coronary artery disease, MACEs were higher in patients treated with insulin compared to patients without insulin therapy [2]. The study published by Jain et al. also showed patients with ITDM to have significantly higher MACEs, all-cause mortality, cardiac death as well as a significantly greater rate of target vessel failure compared to those diabetic patients not on insulin therapy after PCI [15]. Their study also showed no significant difference in definite and probable ST between ITDM and NITDM. Moreover, Bundhun et al. recently confirmed that insulin therapy was associated with significantly higher adverse clinical outcomes compared to NITDM whether during a short or long term follow up period [19]. Their analysis showed results with very low heterogeneity whereby mortality, MACEs, and revascularization were significantly higher in the ITDM group compared to the NITDM group. However, other studies suggested factors such as female gender, insulin resistance, coronary plaque vulnerability and post challenge hyperglycemia to be responsible for these adverse clinical outcomes following PCI [20–23]. However, this current study mainly focused on NITDM and NDM.

No significant difference in ST was observed between NITDM and NDM even if the percentage of ST among NITDM was higher. It should be noted that NITDM could be at an earlier stage of disease without severely being affected by diabetic complications. However, Ford et al. also showed a significantly improved trend in cardiovascular diseases among a population of United States between the years 1999 to 2000 and the years 2009 to 2010 respectively [24]. 7,751 patients from the National Health and Nutrition Examination Survey were used whereby a mean 10-year prediction of coronary heart disease was 7.2 and 6.5% during the years 1999 to 2000 and 2009 to 2010 respectively, and 9.2 and 8.7% for cardiovascular diseases during the same time period respectively. In addition, Gregg et al. [25] also showed a decline in the rate of complications due to T2DM between the years 1990 to 2010 which could be another reason due to which, ST did not differ significantly between NITDM and NDM in this current analysis.

This study is new in several ways. First of all, it is the first meta-analysis systematically comparing the adverse clinical outcomes between NITDM and NDM patients following PCI. Several studies compared T2DM with NDM, ITDM with NITDM, but this is the first meta-analysis comparing NITDM with NDM following PCI. The short-term and long-term follow up periods analyzed in this particular study have also added novelty to this research. Moreover, a larger number of patients obtained from randomized trials and observational studies were included in this analysis further contributing to its novelty.

### Limitations

This study also has limitations. First of all, due to the smaller number of patients analyzed, a robust result might not be expected. Moreover, in one study, all cause-death was not reported. However, data for cardiac death was considered and included in the subgroup analyzing all-cause mortality. This might have an effect on the result of this analysis. In addition, several types of ST were considered and analyzed altogether without relying a particular definition. To be more clear, ST defined according to protocol and ST defined according to academic research consortium (ARC) were combined and analyzed. This could have contributed to another main limitation of this study. Furthermore, PCI and anti-platelet therapy have evolved significantly during the last 5 years with new technologies, and new platelet inhibitors preventing ST and resulting in a lower level of complications among patients with T2DM. Ignoring this major consideration could have had a major effect on the result of this study further contributing to its limitations.

## Conclusion

According to this analysis, short and long term MACEs and mortality were significantly higher in patients with NITDM compared to patients without diabetes following PCI. Revascularization also significantly favored patients without T2DM. However, stent thrombosis was not significantly different.

## Abbreviations

ITDM: Insulin-treated diabetes mellitus
MACEs: Major adverse cardiac events
NDM: Non-diabetes mellitus
NITDM: Non-insulin treated diabetes mellitus
PCI: Percutaneous coronary intervention
ST: Stent thrombosis

## References

[1] Mathew V, Gersh BJ, Williams BA (2004). Outcomes in patients with diabetes mellitus undergoing percutaneous coronary intervention in the current era: a report from the Prevention of REStenosis with Tranilast and its Outcomes (PRESTO) trial. Circulation.

[2] Dangas GD, Farkouh ME, Sleeper LA (2014). Long-term outcome of PCI versus CABG in insulin and non-insulin-treated diabetic patients: results from the FREEDOM trial. J Am Coll Cardiol.

[3] Higgins JP (2008). Assessing risk of bias in included studies. Cochrane handbook for systematic reviews of interventions.

[4] Liberati A, Altman DG, Tetzlaff J (2009). The PRISMA statement for reporting systematic reviews and meta-analyses of studies that evaluate healthcare interventions: explanation and elaboration. BMJ.

[5] Kappetein AP, Head SJ, Morice MC (2013). Treatment of complex coronary artery disease in patients with diabetes: 5-year results comparing outcomes of bypass surgery and percutaneous coronary intervention in the SYNTAX trial. Eur J Cardiothorac Surg.

[6] Kereiakes DJ, Cutlip DE, Applegate RJ (2010). Outcomes in diabetic and nondiabetic patients treated with everolimus- or paclitaxel-eluting stents: results from the SPIRIT IV clinical trial (Clinical Evaluation of the XIENCE V Everolimus Eluting Coronary Stent System). J Am Coll Cardiol.

[7] Kirtane AJ, Ellis SG, Dawkins KD (2008). Paclitaxel-eluting coronary stents in patients with diabetes mellitus: pooled analysis from 5 randomized trials. J Am Coll Cardiol.

[8] Kirtane AJ, Patel R, O'Shaughnessy C (2009). Clinical and angiographic outcomes in diabetics from the ENDEAVOR IV trial: randomized comparison of zotarolimus- and paclitaxel-eluting stents in patients with coronary artery disease. JACC Cardiovasc Interv.

[9] Kumar R, Lee TT, Jeremias A (2007). Comparison of outcomes using sirolimus-eluting stenting in diabetic versus nondiabetic patients with comparison of insulin versus non-insulin therapy in the diabetic patients. Am J Cardiol.

[10] Stone GW, Kedhi E, Kereiakes DJ (2011). Differential clinical responses to everolimus-eluting and Paclitaxel-eluting coronary stents in patients with and without diabetes mellitus. Circulation.

[11] Tada T, Kimura T, Morimoto T (2011). Comparison of 3-year clinical outcomes after sirolimus-eluting stent implantation among insulin-treated diabetic, non-insulin-treated diabetic, and non-diabetic patients from j-Cypher registry. Am J Cardiol.

[12] Thukkani AK, Agrawal K, Prince L (2015). Long-Term Outcomes in Patients With Diabetes Mellitus Related to Prolonging Clopidogrel More Than 12 Months After Coronary Stenting. J Am Coll Cardiol.

[13] Witzenbichler B, Mehran R, Guagliumi G (2011). Impact of diabetes mellitus on the safety and effectiveness of bivalirudin in patients with acute myocardial infarction undergoing primary angioplasty: analysis from the HORIZONS-AMI (Harmonizing Outcomes with RevasculariZatiON and Stents in Acute Myocardial Infarction) trial. JACC Cardiovasc Interv.

[14] Massalha S, Luria L, Kerner A (2016). Heart failure in patients with diabetes undergoing primary percutaneous coronary intervention. Eur Heart J Acute Cardiovasc Care.

[15] Jain AK, Lotan C, Meredith IT (2010). Twelve-month outcomes in patients with diabetes implanted with a zotarolimus-eluting stent: results from the E-Five Registry. Heart.

[16] Silber S, Serruys PW, Leon MB (2013). Clinical outcome of patients with and without diabetes mellitus after percutaneous coronary intervention with the resolute zotarolimus-eluting stent: 2-year results from the prospectively pooled analysis of the international global RESOLUTE program. JACC Cardiovasc Interv.

[17] Jensen LO, Thayssen P, Junker A (2012). Comparison of outcomes in patients with versus without diabetes mellitus after revascularization with everolimus- and sirolimus-eluting stents (from the SORT OUT IV trial). Am J Cardiol.

[18] Jensen LO, Maeng M, Thayssen P (2010). Long-term outcomes after percutaneous coronary intervention in patients with and without diabetes mellitus in Western Denmark. Am J Cardiol.

[19] Bundhun PK, Li N, Chen MH (2015). Adverse cardiovascular outcomes between insulin-treated and non-insulin treated diabetic patients after percutaneous coronary intervention: a systematic review and meta-analysis. Cardiovasc Diabetol.

[20] Trifunovic D, Stankovic S, Sobic-Saranovic D (2014). Acute insulin resistance in ST-segment elevation myocardial infarction in non-diabetic patients is associated with incomplete myocardial reperfusion and impaired coronary microcirculatory function. Cardiovasc Diabetol.

[21] Iguchi T, Hasegawa T, Otsuka K (2014). Insulin resistance is associated with coronary plaque vulnerability: insight from optical coherence tomography analysis. Eur Heart J Cardiovasc Imaging.

[22] Lopez-de-Andres A, Jimenez-Garcia R, Hernandez-Barrera V (2014). National trends in utilization and outcomes of coronary revascularization procedures among people with and without type 2 diabetes in Spain (2001–2011). Cardiovasc Diabetol.

[23] Kuramitsu S, Yokoi H, Domei T (2013). Impact of post-challenge hyperglycemia on clinical outcomes in Japanese patients with stable angina undergoing percutaneous coronary intervention. Cardiovasc Diabetol.

[24] Ford ES (2013). Trends in predicted 10-year risk of coronary heart disease and cardiovascular disease among U.S. adults from 1999 to 2010. J Am Coll Cardiol.

[25] Gregg EW, Li Y, Wang J, Burrows NR, Ali MK, Rolka D, Williams DE, Geiss L (2014). Changes in diabetes-related complications in the United States, 1990–2010. N Engl J Med.

